# Posterior Urethral Strictures

**DOI:** 10.1155/2015/628107

**Published:** 2015-11-24

**Authors:** Joel Gelman, Eric S. Wisenbaugh

**Affiliations:** University of California, Irvine, 333 City Boulevard West, Suite 1240, Orange, CA 92868, USA

## Abstract

Pelvic fracture urethral injuries are typically partial and more often complete disruptions of the most proximal bulbar and distal membranous urethra. Emergency management includes suprapubic tube placement. Subsequent primary realignment to place a urethral catheter remains a controversial topic, but what is not controversial is that when there is the development of a stricture (which is usually obliterative with a distraction defect) after suprapubic tube placement or urethral catheter removal, the standard of care is delayed urethral reconstruction with excision and primary anastomosis. This paper reviews the management of patients who suffer pelvic fracture urethral injuries and the techniques of preoperative urethral imaging and subsequent posterior urethroplasty.

## 1. Introduction

Pelvic fracture trauma in males, often secondary to motor vehicle trauma or pelvic crush injuries, can be associated with injuries to the posterior urethra, especially where there is pubic symphysis diastasis or there are displaced inferomedial pubic bone fractures [1]. The term “prostatomembranous disruption” is often used to describe these injuries, and this terminology suggests that the transection occurs at the junction of the prostatic and membranous portions of the posterior urethra. However, more recent studies, including an autopsy review of male patients who sustained pelvic fracture related urethral injuries and died of associated multiple trauma, revealed that the injuries are generally membranous and distal to the urogenital diaphragm [2]. There can be proximal or distal extension, but the injury generally remains distal to the verumontanum of the prostate. As long as the bladder neck remains intact, continence should be maintained in these patients after repair. Additionally, in many patients, there also remains a significant rhabdosphincter contribution, as demonstrated by video-urodynamic testing after reconstruction [3].

The classic sign of urethral injury in a patient with a pelvic fracture is blood at the urethral meatus, but other symptoms such as bladder distension, inability to void, and perineal hematoma should raise a high index of suspicion as well. Older texts emphasize the finding of a high riding prostate on digital rectal examination, but this is not a reliable finding on physical exam.

## 2. Initial Evaluation and Management

**Figure 1:**
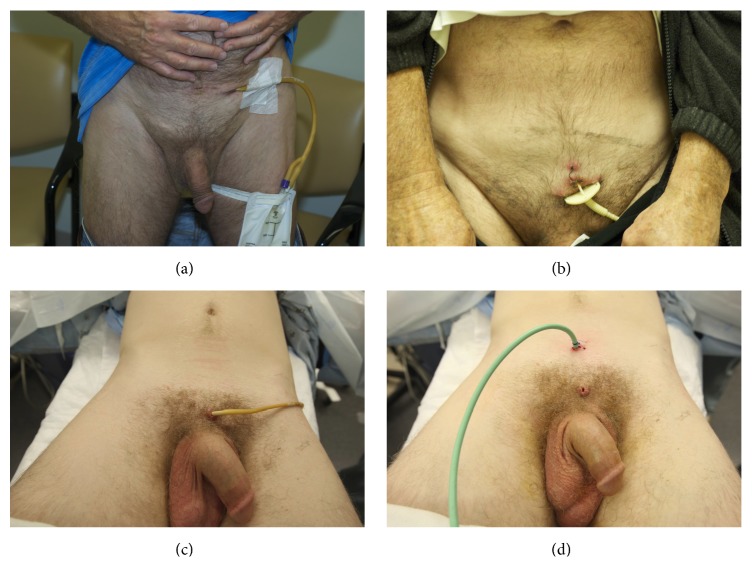
(a) Laterally placed suprapubic tube. (b) Small “pigtail” catheter of inadequate caliber. (c) Suprapubic tube placed below the ideal location. (d) Suprapubic tube repositioned midline 2-finger breadth above the midline pubic symphysis.

A retrograde urethrogram (RUG) is indicated when a urethral injury is suspected and will typically reveal significant extravasation due to a partial tear or, more often, a complete disruption (Figure 1). Initial management should be placement of a suprapubic tube as the most effective and immediate way to drain the bladder. The ideal suprapubic tube is no less than 16 French in size and positioned in the midline 2-finger breadth above the pubic symphysis. Subsequently, options include primary realignment or suprapubic diversion for several months followed by posterior urethroplasty.

The purpose of primary realignment is to approximate the severed ends of the urethra to potentially avoid subsequent stricture formation. Historically, this was performed through an open approach with an attempt at immediate repair. This procedure was mostly abandoned due to the prohibitively high rates of erectile dysfunction and incontinence that resulted compared to those who underwent delayed repair [4]. The advancement of endoscopic technology, however, has allowed primary endoscopic realignment (PER) of the urethra without the potentially damaging extensive manipulation that was required for immediate open repair and is now attempted routinely in some centers.

Stricture formation can potentially be avoided with this approach. However stricture rates remain high after PER and it is essential that these patients be followed in the long term. A recent meta-analysis reported a 49% rate of stricture formation after PER, yet this is likely an underestimate as the literature consists of mostly small case series that are retrospective and have variable follow-up that does not always include cystoscopy to confirm patency [5].

While sometimes successful, PER can have unintended consequences when strictures are not treated appropriately. In a recent study, the mean time to definitive resolution of stenosis was dramatically longer in patients who underwent PER (122 months versus 6 months) because they underwent multiple endoscopic interventions without resolution of their stenosis [6]. Repeated, not only are unsuccessful interventions costly to the healthcare system, but they can also expose the patient to painful self-dilations or office dilations as well as the potential for acute urinary retention requiring emergency management.

The purpose of limiting the immediate management to placement of a suprapubic tube is to allow a successful, definitive repair once the tissues have had time to heal, typically after 3 months. Placement of a suprapubic tube can easily be accomplished in any trauma center without the need for immediate reconstructive expertise, and the patient can subsequently be referred for further management. In this situation, posterior urethroplasty is highly successful, with a patency rate of 97.6% at our own institution.

Although the benefits of primary realignment are the subject of controversy, it should be emphasized that when a stricture develops after primary realignment, the subsequent management is not controversial. The best approach is suprapubic tube urinary diversion for several months followed by urethral reconstruction. Management with dilations, urethrotomies, and/or self-catheterization should not be advised in an attempt to avoid open surgery as these options manage a chronic problem whereas a properly performed urethroplasty is almost always curative.

## 3. Preoperative Planning

### 3.1. Three-Month Delay

We wait 3 months from the time of injury or catheter removal in cases of failed primary realignment before performing urethroplasty to allow time for the initial extravasation to heal, hematoma to resolve, and the extent of the injury to become clearly defined. It has been shown that, after manipulation, several months of “urethral rest” is required before anterior urethral strictures become clearly defined [7]. When there is a pelvic fracture associated injury to the posterior urethra, initial imaging reveals extravasation, whereas imaging 3 months after injury typically confirms no extravasation and clear delineation of the location and length of the defect. Recent publications indicate that the delay is often a minimum of 3–6 months. However, the interval between initial injury and urethroplasty can exceed one year when there are associated injuries [8–12].

### 3.2. Suprapubic Tubes

Although the ideal suprapubic tube is at least 16 Fr, midline, and well above the midline pubic symphysis, patients are often initially managed with tubes that are far lateral to the midline or just above the symphysis. In some cases, very small caliber “pigtail” catheters are placed (Figure 1). Small caliber pigtail catheters are especially prone to encrustation and ultimately urinary retention. Moreover, catheters placed just above the symphysis are more uncomfortable than catheters placed in a higher position away from the bone. When patients are referred for posterior urethral reconstruction and have tubes of inadequate caliber, or if the tube is not in the ideal position, it is our preference to percutaneously place a new 16 Fr tube. This is generally done as soon as possible when the caliber is small and in no less than 1 month prior to urethroplasty so there will be an established stable tract at the time of surgery.

The main benefit of having the suprapubic tube midline in the ideal location with an established tract is that this facilitates the surgery and prevents the need for a temporary vesicostomy. During posterior urethroplasty with the patient in the lithotomy position, after perineal exposure is achieved and the urethra is transected, a metal sound is generally advanced through the established tract, and perineal dissection proceeds towards the tip of the sound until the sound can be seen and advanced into the perineum. When the caliber of the tract is inadequate, sounds will not advance without dilation at the time of the surgery. This can be associated with bleeding and compromise of the tract. When the tube is just above the bone, a very acute angle is needed to advance the sound through the bladder neck. Moreover, when the tract is lateral to the midline, the rigid sound cannot be reliably advanced medially towards the midline bladder neck and then distally along the posterior urethra. One option is to create a temporary vesicostomy. However, this adds considerable time and morbidity to the reconstructive surgery and therefore this is not our preference.

### 3.3. Preoperative Urethral Imaging and Cystoscopy

**Figure 2:**
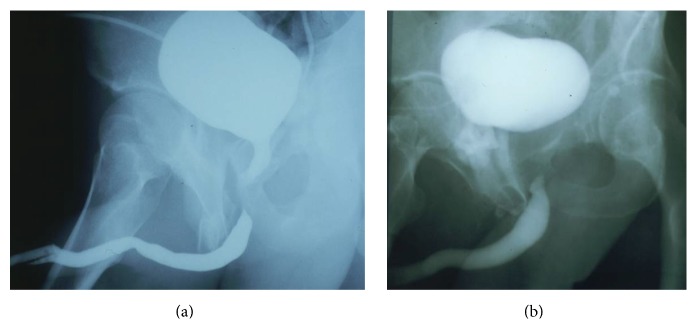
(a) After the bladder is filled with contrast through the suprapubic tube, a RUG is performed as the patient is asked to attempt to void. If the bladder neck opens, contrast fills the prostatic urethra, and the membranous urethral defect is seen. (b) When the bladder neck does not open, the length of the defect cannot be determined accurately.

Prior to definitive urethral reconstruction, urethroscopy, antegrade cystoscopy, and simultaneous antegrade cystourethrogram and retrograde urethrogram (RUG) provide a definitive diagnosis of the exact length and location of the defect. One common imaging technique is for the bladder to be filled with contrast by gravity through the suprapubic tube and for a RUG to be performed as the patient is asked to Valsalva and attempt to void. This attempt to void can open the bladder neck and allow filling of the posterior urethra proximal to the obliteration, and the length of the defect will be determined (Figure 2(a)). However, in many cases, the patient cannot relax to void when the urethra is obliterated and contrast is being injected through the penis. When the bladder neck is intact, the appearance will be as shown (Figure 2(b)). The distance between the bladder and the distal end of the defect is not the length of the distraction defect because the prostatic urethra is not visualized. In a recent study where the goal was to determine if the type of urethroplasty could be predicted based on certain features from the preoperative imaging, 38% of the 100 study patients evaluated with a Valsalva cystourethrogram and RUG were excluded because there was no visualization of the urethra below the bladder neck [7].

Our preferred approach is to first perform antegrade cystoscopy with the patient prepped and draped in the oblique position after a 14 × 17 scout film is obtained to confirm proper position and penetration. Antegrade cystoscopy is important to inspect for bladder stones that may need to be removed preoperatively and also provides assessment of the bladder neck. An open bladder neck at rest suggests that there may be an increased incidence of incontinence subsequent to urethral reconstruction. Iselin and Webster identified 15 patients who sustained pelvic fracture urethral injuries and had an open bladder neck at rest [13]. Six were continent and 8 were incontinent after urethroplasty. However, MacDiarmid et al. identified 4 patients that had an open bladder neck at rest and all of these patients were continent after surgery [14]. Although some surgeons occasionally perform bladder neck reconstruction at the time of posterior urethroplasty [15], most do not feel this is necessary given the observation that an open bladder neck at rest does not reliably predict postoperative incontinence. When we observe an open bladder neck at rest, the patient is counseled that there may be an increased incidence of postoperative incontinence, but this finding does not influence our management.

### 3.4. Preoperative Urethral Evaluation: Urethral Imaging

**Figure 3:**
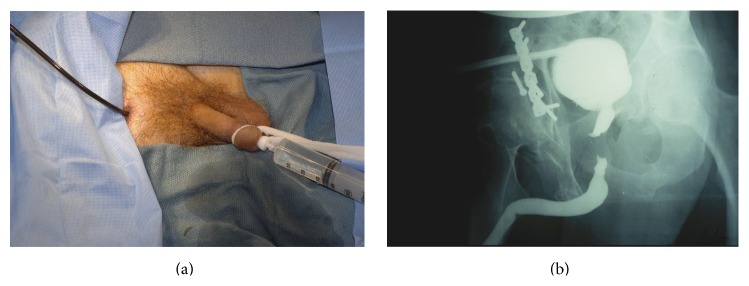
(a) A RUG is performed as contrast is simultaneously injected into the posterior urethra through the flexible cystoscope, with the tip in the distal prostatic urethra. (b) Imaging accurately demonstrating the length and location of the defect.

**Figure 4:**
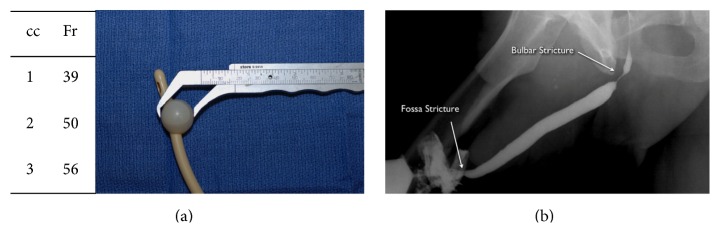
(a) Catheter balloon inflation with only 1–3 cc of air or fluid is associated with balloon inflation well beyond the normal caliber of the normal fossa navicularis. (b) Repeat RUG demonstrating, in addition to the previously seen bulbar stricture, a new fossa navicularis stricture that developed after a RUG was performed using fossa balloon inflation technique.

Once the scope is advanced through the bladder neck, the location of the proximal aspect of the injury is noted, and this is almost always distal to the verumontanum of the prostate within the membranous urethra. With the tip of the scope at the level of the obliteration, full-strength contrast is injected, which will then backfill the posterior urethra and bladder. Simultaneously, a RUG is performed. Our preferred technique for performing a RUG is to place a gauze around the coronal sulcus to place the penis on stretch and inject contrast through a cone-shaped Taylor adaptor (Cook Urological) connected to a 60 cc syringe filled with full-strength contrast (Figure 3). Many published textbooks advocate the advancement of a catheter into the fossa navicularis and inflation of the balloon with 1–3 cc of contrast to form a seal. However, the balloon caliber of catheters of several different sizes when inflated with only 2 cc of fluid or air is approximately 59 French and the normal caliber of the adult anterior urethra is approximately 30 French except at the level of the urethral meatus and fossa navicularis where the caliber is approximately 24 French (Figure 4(a)). Therefore, the balloon will dilate the normal distal anterior urethra, which can be associated with considerable pain and even stricture disease of the fossa navicularis. We have seen patients referred for strictures initially limited to the bulbar urethra who then developed narrow caliber fossa strictures after undergoing painful urethral imaging where the technique included balloon inflation within the fossa navicularis (Figure 4(b)).

Simultaneous antegrade and retrograde imaging and endoscopy performed with proper technique will clearly define the exact length and location of the defect. Other imaging modalities that can be used include MRI and ultrasound [16]. However, we have never found an indication to perform these additional tests. Fluoroscopy offers the advantage of dynamic real time imaging. However, disadvantages include a reduced field of view and decreased resolution compared to conventional radiographs. We prefer flat plate imaging using digital cassettes that can be digitized and stored electronically and also printed on 14 × 17 film. Although magnification and positioning can influence the scale, we have observed that the length of the obliteration measured directly on the film very accurately corresponds to the length of the defect at the time of surgery. Most defects are 1 to 3 cm in length.

**Figure 5:**
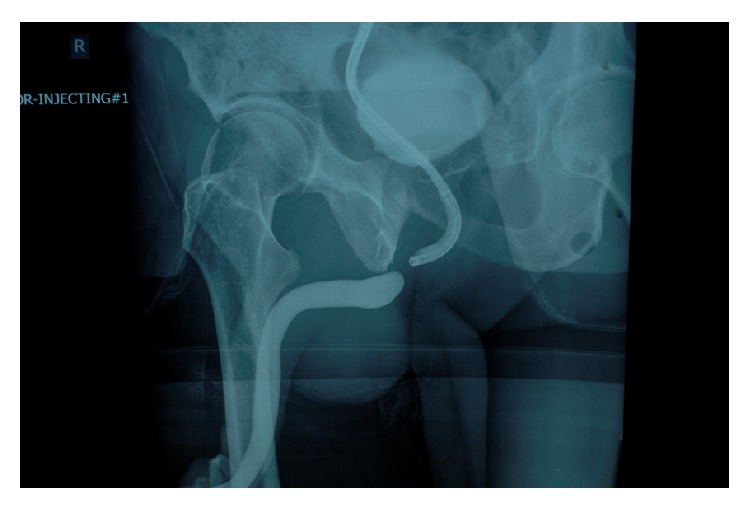
Simultaneous antegrade and retrograde urethral imaging demonstrating a bulbar urethral obliteration, further confirmed with antegrade cystoscopy.

Defining the exact location of a stricture is also critical to management. Although pelvic fracture trauma typically injures the posterior urethra, if there is also straddle trauma at the time of the pelvic fracture, the injury can be to the bulbar urethra, which changes the treatment strategy. For example, a man who sustained pelvic fracture trauma during a race car accident was found to have significant extravasation on a RUG on the day of the injury and managed with a laterally placed suprapubic tube. Delayed imaging and antegrade cystoscopy confirmed a proximal bulbar urethral defect and a normal membranous urethra (Figure 5). Although both traumatic proximal bulbar and membranous disruptions are managed with excision and primary anastomosis, bulbar urethroplasty does not require antegrade access to facilitate identification of the patent proximal segment. If the injury was membranous, then a new midline suprapubic tube would have been placed to facilitate subsequent antegrade access to the proximal segment at the time of posterior urethroplasty. However, since antegrade access is not required for bulbar urethroplasty, the placement of a new midline tube was not required.

### 3.5. Preoperative Vascular Evaluation

**Figure 6:**
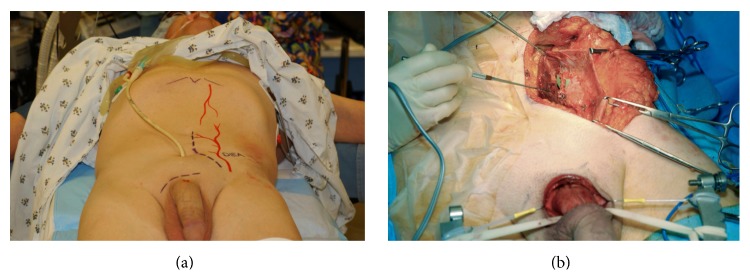
Inferior epigastric artery to dorsal artery penile revascularization, shown subsequent to skin marking (a) and during surgery (b).

The anterior urethra has a dual blood supply, with an additional minor contribution provided by perforating vessels between the corpora cavernosa and the corpus spongiosum. The bulbar arteries enter the corpus spongiosum at the level of the most proximal bulbar urethra and provide antegrade flow to the corpus spongiosum of the anterior urethra. In addition, the dorsal arteries course within the neurovascular structures along the dorsal aspect of the penis superficial to the corporal bodies and supply the glans penis, which is the distal expansion of the corpus spongiosum. This provides a secondary blood supply to the anterior urethra as the blood courses in retrograde fashion along the corpus spongiosum. When the urethra is completely transected at the departure of the anterior urethra, any patent bulbar arteries are ligated or cauterized. The anterior urethra then survives as a flap based on the retrograde dorsal artery contribution in addition to perforating vessels. Although unpublished, it has been observed by several reconstructive urologists that, in rare cases, long segment bulbar strictures developed as an ischemic complication of posterior urethroplasty. The mechanism of the ischemic stenosis was presumed to be compromised of the bulbar artery supply during surgery in patients who suffered perineal trauma that compromised dorsal artery supply. We have observed cases of ischemic stenosis in patients with hypospadias who developed discreet bulbar strictures and were treated with a urethral stent [17]. Prior to stent placement, these patients were noted on urethroscopy to have a normal caliber anterior urethra distal to the bulbar stricture. Subsequent to stent placement, they developed severe panurethral disease. This makes sense anatomically as hypospadias and corrective surgery are associated with a compromise of the corpus spongiosum distally and the associated retrograde blood supply to the more proximal anterior urethra. These patients were likely “bulbar dependent,” and stent expansion compromised the antegrade bulbar artery flow distal to the stent. Therefore, in addition to antegrade cystoscopy and contrast imaging, we perform a preoperative vascular evaluation to identify patients who have severe arterial inflow compromise to both dorsal arteries and perform penile revascularization prior to urethral reconstruction in selected cases. Penile revascularization provides a microvascular anastomosis of the inferior epigastric artery to the dorsal artery of the penis (Figure 6).

Erectile dysfunction and pudendal vascular injuries are highly associated with pelvic fracture urethral disruptions. A recent meta-analysis revealed that 34% of all patients with pelvic fracture urethral injury developed erectile dysfunction, even prior to any treatment other than suprapubic tube placemat [18]. In a study by Shenfeld et al., 25 patients who sustained traumatic posterior urethral disruptions were evaluated with nocturnal penile tumescence testing [19]. Eighteen patients (72%) were found to have erectile dysfunction, and these patients underwent a penile duplex with pharmacologic erection that revealed arterial inflow impairment in 5/18 patients. The remaining patients were considered to have a neurogenic etiology of their erectile dysfunction. Davies et al. performed a penile duplex testing on 56 men who sustained posterior urethral disruptions and identified 25 men with vascular compromise. These patients underwent arteriogram. Twenty-one had reconstitution of one or both pudendals, and 4 did not. These 4 patients underwent revascularization prior to urethral reconstruction, and no patient developed ischemic stenosis after surgery [20]. A limitation of this study is that it is not known if any of these patients would have developed stenosis if reconstruction had been performed without prior revascularization. This is an area of controversy. However, we believe vascular testing and revascularization in selected cases are justified based on the anatomic principals and the available data. Moreover, revascularization will often successfully treat the erectile dysfunction associated with pelvic fracture injuries [21].

### 3.6. Posterior Urethral Reconstruction: Preparation and Patient Positioning

Prior to definitive repair, patients are placed in high lithotomy during a physical examination to assess hip flexion and ability to tolerate this position. Some patients may have unresolved back or other orthopedic problems, which may then be exacerbated by prolonged lithotomy positioning. In our series of 85 patients, the longest delay was 19 months. This patient had severe compromise of hip flexion that persisted more than 12 months after the injury. With ongoing physical therapy, mobility returned to normal, and positioning was safely accomplished without compromise. A urine culture is sent the week prior to surgery. The specimen is obtained by clamping the suprapubic tube and then unclamping the tube 20 minutes later over a specimen container. The sample is then obtained directly from the suprapubic tube and not the drainage bag. Any mixed growth is separately cultured. Patients are admitted the day prior to surgery for dual coverage antibiotics. Our protocol is to administer piperacillin/tazobactam and tobramycin but adjust the antibiotics if indicated based on the culture result. To date, no patient suffered the complication of a perineal infection, which can be associated with urethral compromise and stricture development.

Although some reconstructive urologists prefer a low lithotomy position, we prefer the exposure of exaggerated lithotomy. However, this position can be associated with severe complications including neuropraxia, compartment syndrome, and rhabdomyolysis [22–24]. Fortunately, neuropraxia is usually not permanent. Sensory deficits are more common than motor impairment, and the risk of a positioning complication is related to the time in lithotomy. One form of exaggerated lithotomy, often used for perineal prostatectomy, places hips under considerable flexion so that the thighs are parallel to the back and the floor. In a study by Holzbeierlein et al., of 111 men who underwent a radical perineal prostatectomy in this extreme lithotomy position with a mean duration of less than 3 hours, 23 (21%) suffered a positioning complication. Of these 23 patients, 17 had symptoms at the time of discharge, and 6 required physical therapy support for ambulation [25].

**Figure 7:**
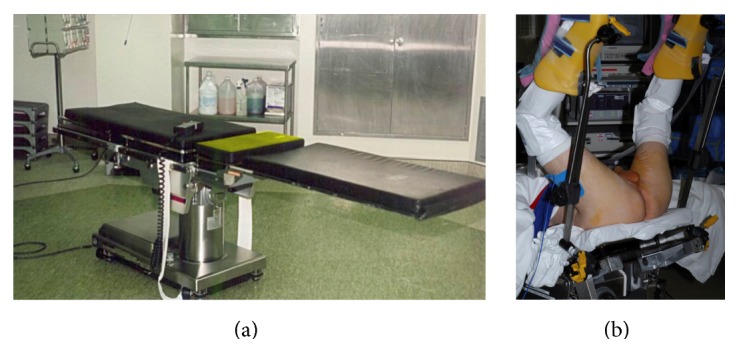
(a) Modified Skytron Custom 6000 Surgical Table with pelvic tilt mechanism (highlighted in yellow). (b) Patient positioned in exaggerated lithotomy.

We use Skytron Custom 6000 Table modified by Jordan to offer an electronic pelvic tilt mechanism to cradle the pelvis as an alternative to raising the buttocks and placing a beanbag support (Figure 7(a)). In addition, stirrups are modified to provide additional extension so that hip and knee flexion is reduced. Foam padding is placed along the dorsal feet and anterior legs to evenly distribute the pressure (Figure 7(b)). We previously used gel pads but found that use of the softer foam pads reduced the incidence of temporary (24–28 hours) dorsal foot numbness. Extreme flexion of the hips and knees is avoided, and the boots are tilted so that there is no pressure on the calves.

### 3.7. Posterior Urethral Reconstruction: Surgical Technique

#### 3.7.1. Exposure

**Figure 8:**
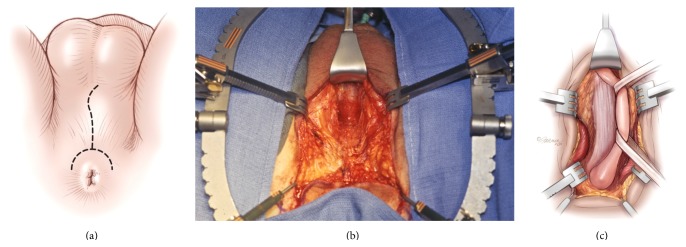
(a) Lambda incision with the patient in the exaggerated lithotomy position. (b) Jordan-Simpson perineal retractor is used to facilitate exposure of the corpus spongiosum. (c) The corpus spongiosum is circumferentially mobilized along the bulbar urethra.

A midline perineal incision is one option. We prefer an inverted “Y” shaped lambda incision to obtain generous exposure (Figure 8(a)). This is carried medial to the ischial tuberosities posteriorly and along the median scrotal raphe. Dissection then proceeds sharply through the subcutaneous fat longitudinally along the midline until the bulbospongiosus muscle is encountered. The Jordan-Simpson perineal retractor is used to facilitate exposure as shown (Figure 8(b)). Although other retractors are commercially available such as the Lone Star retractor, advantages of the Jordan retractor include the fixation of the ring and the ability to use a variety of different specialized blades in addition to the hooks used in the Lone Star system. In addition, tilt ratchets facilitate lateral retraction to facilitate exposure. The bulbospongiosus muscle is then divided and retracted laterally to expose the bulb. The bulb is detached from the perineal body and we find that the use of a bipolar cautery facilitates this dissection and maintains hemostasis to the extent that suction is seldom required. The urethra is then circumferentially mobilized from the penoscrotal junction distally to the departure of the anterior urethra proximally (Figure 8(c)). This is done sharply without the use of right angle clamps, which can tear the corpus spongiosum. The bulbar arteries are transected and cauterized if patent.

Several recent papers describe bulbar artery sparing anastomotic anterior urethroplasty [26, 27]. Although the use of artery sparing surgery during posterior urethral reconstruction has not been published, an abstract recently presented described the successful use of this technique in 9 patients [28]. Intraoperative ultrasound was performed, and the artery with the strongest signal was preserved. No patient developed a recurrent stricture with a mean follow-up of 10 months. This may possibly represent a future modification of operative technique.

#### 3.7.2. Proximal Exposure and Scar Excision

Once the urethra has been adequately mobilized, it is transected at the distal aspect of the defect, which can be accurately located intraoperatively with the use of a 16 Fr catheter or bougie á boule. Subsequently, unless preoperative imaging suggests very short segment obliteration, we routinely separate the corporal bodies at the level of the triangular ligament and retract them laterally to improve proximal exposure and facilitate excision of the scar tissue, which is generally whitish in color and firm.

**Figure 9:**
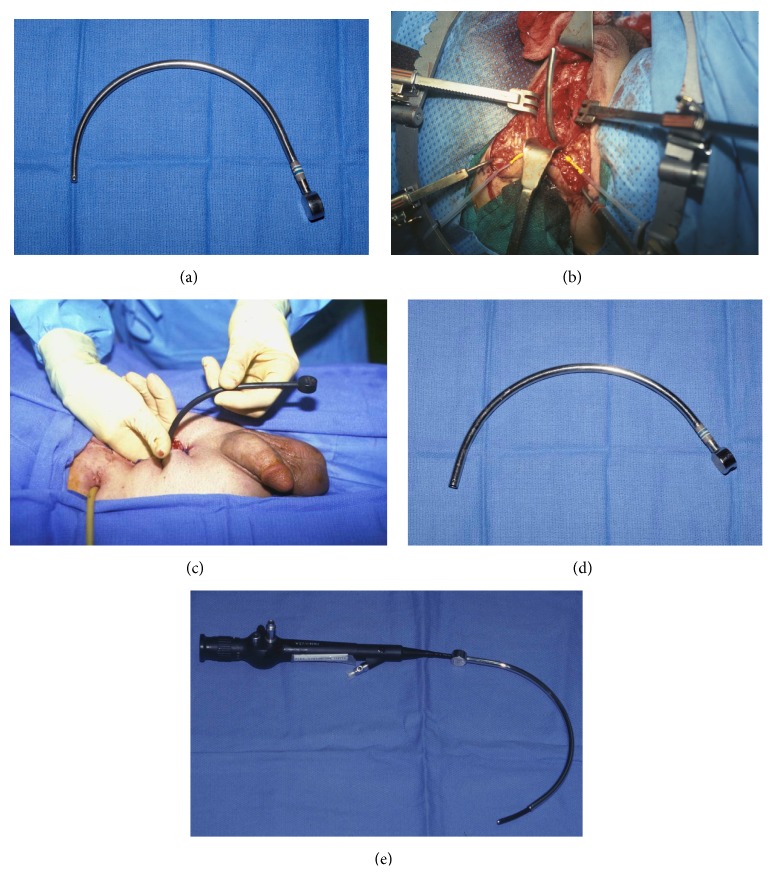
(a) Solid Haygrove sound. (b) After dissection of the obliterative scar, the tip of the sound (placed through the suprapubic tract) can then be advanced through the patent proximal urethra into the perineum. (c) Temporary vesicostomy in a patient with a laterally placed suprapubic tube. (d) Gelman visualizing posterior urethral sound. (e) Flexible scope advanced through the hollow visualizing sound.

The suprapubic tube is then removed and a curved metal sound is advanced through the established tract into the bladder and then through the bladder neck, guided by feel, until the impulse of the tip of the sound can be palpated in the perineum as the sound is manipulated. This guides the dissection in the appropriate direction towards the patent proximal urethra. One option is to advance a Van Buren sound. Although these instruments are often readily available and familiar to most urologists, the fact that the instrument is curved only at the tip and tapered to a more pointed tip relative to the shaft of the instrument renders these instruments poorly suited to use in posterior urethroplasty, especially when an exaggerated lithotomy position is used. However, the semicircular Haygrove sound is designed to best follow the path from the suprapubic access to the membranous urethra (Figures 9(a) and 9(b)). The tip is curved and smooth, and the caliber is not greater than 16–18 Fr and, therefore, no tract dilation is required if the indwelling suprapubic tube was 16 Fr.

There are cases, however, when the tip of the sound may not be palpable. This may be due to the presence of very dense scar or malposition of the sound. This presents a significant challenge because if dissection proceeds in the wrong direction, what is entered may be the bladder or the posterior urethra proximal to the distal aspect of the patent urethra. This could essentially “bypass” the bladder neck and may lead to severe postoperative incontinence. This is a major limitation of using a solid sound that is guided blindly. A flexible cystoscope can be used in these cases, but since the active scope deflection is limited only to the tip of the scope, it may be difficult to advance the tip of the scope to the proper position, especially when the patient is in high lithotomy and the surgeon is positioned at the level of the perineum. To prevent the possibility of false passages, some surgeons perform rigid antegrade cystoscopy before the patient is prepped and draped in the exaggerated lithotomy position, advance the scope through the bladder neck and prostatic urethra, and then palpate the perineum to determine if the tip of the scope is palpable or not [29]. When the tip of the scope is not palpable, or if the suprapubic tube is laterally located, a temporary vesicostomy is created prior to lithotomy positioning and then taken down subsequent to the completion of the repair (Figure 9(c)). It was determined that the creation of the vesicostomy allows the surgeon to palpably identify the bladder neck before instrumentation of the posterior urethra and that this maneuver eliminates the occurrence of false passages and the misanastomosis of the anterior urethra to sites other than the apical prostatic urethra. While this maneuver can be effective, it adds considerable time and morbidity to the surgery.

It is for this reason that we prefer to always proceed with a midline suprapubic tube, even if this requires placement of a new tube no less than 1 month prior to urethroplasty to allow time for the tract to mature and use a new visualizing sound (Gelman Urethral Sound, CS Surgical) (Figure 9(d)). This sound has a contour similar to the Haygrove sound but is hollow, allowing a flexible cystoscopy to be advanced through the sound (Figure 9(e)). The tip of the sound and/or the tip of the cystoscope can then be directed to the obliteration under direct vision. An additional advantage is that the light from the scope can be seen to further guide the dissection. Prior to the development of the visualizing sound, 2/9 patients at our institution required a temporary vesicostomy at the time of reconstruction. Subsequently, 76 patients (ages 4–77 years) underwent reconstruction (including 6 pediatric patients and 14 patients who had unsuccessful procedures prior to referral), and 0/76 patients required a temporary vesicostomy. In every case, the sound could be directed to the proper position under direct vision. It is our experience that the visualizing posterior urethral sound greatly facilitates the reliable identification and dissection of the proximal segment during posterior urethral reconstruction. With the use of this device, the open dissection can be limited to the perineal exploration, even in pediatric and difficult cases. One disadvantage of the sound is that the outside diameter (OD) is greater than the OD of the solid Haygrove sound, and the solid sound can be manipulated more easily. Therefore, we continue to use the solid sound when the tip can be readily palpated in the perineum. Although the larger diameter hollow sound will not advance as easily through the suprapubic tract when 16–18 Fr indwelling tubes are used prior to surgery, the tip of the flexible cystoscope can be first advanced into the bladder, and the sound can then be advanced over the scope using the scope as the equivalent of a guide wire.

The most complex portion of posterior urethral reconstruction is the proximal exposure and dissection subsequent to transection of the urethra. One option is to sharply incise scar tissue, advance a nasal speculum through the scar, and place “J” shaped sutures through the speculum to initiate the anastomosis [30]. It is our preference to excise the scar tissue until normal healthy tissue is encountered. Supple tissue more readily everts, bringing the mucosa forward from deep within the pelvis during the placement of the first several sutures, and this facilitates the placement of subsequent sutures. Our objective is to achieve the proximal preplacement of 10-12 3-0 absorbable monofilament sutures. We alternate using violet PDS and clear Monocryl to help maintain orientation at the time of the completion of the anastomosis.

#### 3.7.3. Infrapubectomy

In cases where the scar is especially dense and the defect is long, it is possible that the tip of the sound will not be palpable, and the light of the cystoscope will not be seen even when using the visualizing sound. In these cases, scar tissue just below the midline symphysis is excised sharply in a 1-2 cm diameter area. As the dissection extends deep into the pelvis, infrapubectomy is often required to facilitate the proximal scar excision. The corporal bodies, which have already been separated, are retracted laterally exposing the dorsal vein, which is then mobilized and ligated to expose the midline symphysis pubis. Periosteal elevators are then used to sweep the medial crura laterally and free the undersurface of the bone from adherent tissue. Kerrison rongeurs provide controlled bone removal, which widens the exposure and facilitates further proximal dissection. Moreover, the separation of the corpora and infrapubectomy provide a more direct route for the urethra to course, and this facilitates a tension-free repair.

#### 3.7.4. Additional Maneuvers

Some authors have reported that, in addition to distal mobilization, crural separation, and infrapubectomy, supracrural corporal rerouting was required to achieve an acceptable amount of tension in selected cases [31]. This technique appears to be associated with a high rate of restenosis. In a recently published combined series of 142 cases, 4 underwent rerouting and 3 of these patients (75%) developed restenosis [12]. Other surgeons never find supracrural rerouting to be a beneficial maneuver. It is often stated that the objective is a “tension-free” anastomosis. This is not necessary as there is normally a certain amount of innate tension along the corpus spongiosum. It is for this reason that when the urethra is transected, there is generally some retraction of the distal segment. Our goal is not a tension-free anastomosis, but rather an anastomosis without unacceptable tension that would lead to tethering of the penis during erections or separation of the anastomosis. To date, we have never encountered a case where supracrural rerouting was required.

Another option in complex cases where there is a large defect is a transpubic approach [32–34]. This is a technique we have never found necessary, and more recent reports confirm that infrapubectomy generally provides adequate proximal exposure in complex cases [35].

Another tool that has been reported to bridge longer defects is the use of tissue transfer with flaps or grafts [31]. This appears to have been performed mostly in older series, and recent reports do not support the use of or need for tissue transfer. It is fortunate that excision and primary anastomosis can reliably be achieved during posterior urethral reconstruction given that tube flaps and grafts are generally associated with a high failure rate, and the tissues surrounding the membranous urethra deep within the pelvis proximal to the triangular ligament do not represent an excellent bed for graft spread fixation.

#### 3.7.5. Anastomosis

Once the proximal sutures are placed along a widely patent proximal segment surrounded by pink healthy mucosa proximally, and flexible cystoscopy further confirms that the opening is distal to the verumontanum at the appropriate location, the distal segment is dorsally spatulated and calibrated using bougie á boule. The caliber should be greater than 30 Fr. The anastomosis is then completed as a stenting catheter is placed. It is our preference to use a 14 Fr soft silicone catheter. A small round drain is placed deep adjacent to the corpus spongiosum deep to the bulbospongiosus muscle, which is then reapproximated along the ventral midline, and a second flat 7 mm drain is placed superficial to the muscle. The incision is then closed in 2 layers with absorbable suture and a clear dressing is placed. No compressive dressing is required.

### 3.8. Postoperative Care

Our protocol is to maintain the stenting urethral catheter and the suprapubic tube urinary diversion for 3 weeks and then perform a VCUG by removing the stenting catheter, filling the bladder with contrast by gravity installation and then obtaining a film during urination. In the rare case of extravasation, a new stenting catheter is replaced and a repeat study is performed the following week. Other surgeons favor catheter removal without postoperative imaging [36]. In most cases, the force of stream will be excellent, and the suprapubic tube is then removed. If the stream is weak, the tube is plugged and the patient is instructed to unplug the tube at home, if unable to urinate, to check residuals by unplugging the tube after micturition. One possible reason for voiding difficulty is neurogenic bladder dysfunction related to the initial injury, especially if there is associated back trauma. Several months after tube removal, flexible urethroscopy is performed to definitively confirm wide patency of the repair. Patients are then encouraged to have a baseline flow rate and postvoid residual assessment and then to have this repeated annually. There is currently a lack of consensus regarding appropriate follow-up after surgery.

### 3.9. Outcomes

In our series of 85 patients, prior to referral, 17 underwent failed endoscopic treatment and 17 underwent failed open surgery. At the time of surgery, 19 patients underwent infrapubectomy, and no patient required supracrural rerouting. No patient required transfusion, and the only persistent neuropraxia was in one patient who had persistent tingling of the toes that resolved after several months. At the time of urethroscopy 4 months after surgery, 2 patients were noted to have medium caliber narrowing. One of these patients underwent dilation 2 years after surgery and the other was observed and never required treatment. This corresponds to a success rate of 97.6% success, using the strict definition of maintaining durable wide patency of the repair and with no further treatment required.

Other series report a similar success rate for adults, adolescents, and children, and this indicates that a stricture recurrence after a properly performed posterior urethroplasty should be a rare event [35, 37]. Of the patients who presented to our center after failed surgery, the recurrence was often within days or weeks, suggesting that these were technical failures, likely due to inadequate scar excision. Further suggesting that technical inexperience of the surgeon is likely the most common cause of failure is the fact that these patients usually have a successful outcome with the same technique of excisional repair when the revision surgery is performed by a specialist in urethral reconstruction. Published papers from referral centers confirm that when open repair fails, excision and primary anastomosis still remains the procedure of choice, and when properly performed, it offers a very high success rate [38, 39]. In conclusion, delayed posterior urethral disruption injuries are highly amenable to successful reconstruction with excisional posterior urethroplasty via a perineal approach.
